# The antigenicity of SARS-CoV-2 Delta variants aggregated 10 high-frequency mutations in RBD has not changed sufficiently to replace the current vaccine strain

**DOI:** 10.1038/s41392-022-00874-7

**Published:** 2022-01-19

**Authors:** Jiajing Wu, Jianhui Nie, Li Zhang, Hao Song, Yimeng An, Ziteng Liang, Jing Yang, Ruxia Ding, Shuo Liu, Qianqian Li, Tao Li, Zhimin Cui, Mengyi Zhang, Peng He, Youchun Wang, Xiaowang Qu, Zhongyu Hu, Qihui Wang, Weijin Huang

**Affiliations:** 1 Division of HIV/AIDS and Sex-transmitted Virus Vaccines, Institute for Biological Product Control, National Institutes for Food and Drug Control (NIFDC), 102629 Beijing, China; 2 Research Network of Immunity and Health (RNIH), Beijing Institutes of Life Science, Chinese Academy of Sciences, 100101 Beijing, China; 3 CAS Key Laboratory of Pathogenic Microbiology and Immunology, Institute of Microbiology, Center for Influenza Research and Early-warning (CASCIRE), CAS-TWAS Center of Excellence for Emerging Infectious Diseases (CEEID), Chinese Academy of Sciences, 100101 Beijing, China; 4 Division of Hepatitis and Enteric Viral Vaccines, Institute for Biological Product Control, National Institutes for Food and Drug Control (NIFDC) and Key Laboratory of the Ministry of Health for Research on Quality and Standardization of Biotech Products, 102629 Beijing, China; 5 Translational Medicine Institute, First People’s Hospital of Chenzhou, University of South China, Chenzhou, China; 6 CAS Key Laboratory of Pathogenic Microbiology and Immunology, Institute of Microbiology, Chinese Academy of Sciences, 100101 Beijing, China

**Keywords:** Vaccines, Respiratory tract diseases

## Abstract

Emerging SARS-CoV-2 variants are the most serious problem for COVID-19 prophylaxis and treatment. To determine whether the SARS-CoV-2 vaccine strain should be updated following variant emergence like seasonal flu vaccine, the changed degree on antigenicity of SARS-CoV-2 variants and H3N2 flu vaccine strains was compared. The neutralization activities of Alpha, Beta and Gamma variants’ spike protein-immunized sera were analysed against the eight current epidemic variants and 20 possible variants combining the top 10 prevalent RBD mutations based on the Delta variant, which were constructed using pseudotyped viruses. Meanwhile, the neutralization activities of convalescent sera and current inactivated and recombinant protein vaccine-elicited sera were also examined against all possible Delta variants. Eight HA protein-expressing DNAs elicited-animal sera were also tested against eight pseudotyped viruses of H3N2 flu vaccine strains from 2011–2019. Our results indicate that the antigenicity changes of possible Delta variants were mostly within four folds, whereas the antigenicity changes among different H3N2 vaccine strains were approximately 10–100-fold. Structural analysis of the antigenic characterization of the SARS-CoV-2 and H3N2 mutations supports the neutralization results. This study indicates that the antigenicity changes of the current SARS-CoV-2 may not be sufficient to require replacement of the current vaccine strain.

## Introduction

The coronavirus disease 2019 (COVID-19) pandemic, caused by severe acute respiratory syndrome coronavirus 2 (SARS-CoV-2), created an unprecedented global public health emergency after it was first reported in December 2019. By September 2021, the pandemic had spread to 212 countries and regions, with more than 224 million confirmed cases and over 4.6 million deaths (https://covid19.who.int/). SARS-CoV-2 belongs to the genus Beta coronavirus, comprising enveloped, positive sense, single-stranded RNA viruses. The SARS-CoV-2 genome encodes four structural proteins, spike (S), envelope (E), membrane (M), and nucleocapsid (N) proteins.^1,2^ The S protein, located on the surface of viral particles, is the main antigenic target of protective immune responses.^3,4^ Almost all SARS-CoV-2 vaccines in the various stages of development have been based on the S protein, particularly on that of the original strain.

Like other positive-stranded RNA viruses, a high mutation frequency occurs during viral replication because of the error-prone nature of RdRp, which enables the rapid adaptation and evolution of viruses.^5,6^ Although SARS-CoV-2 has a much lower mutation frequency than many other RNA viruses, several variants have appeared since the last quarter of 2020.^7^ The World Health Organization (WHO) has established global networks to monitor SARS-CoV-2 variants and has defined variants of interest (VOIs) and variants of concern (VOCs) to inform the ongoing response to the COVID-19 pandemic. On August 12, 2021, there were four VOCs and four VOIs, most of which had altered antigenicity compared with that of the original SARS-CoV-2. Among the VOCs and VOIs, the Alpha variant (B.1.1.7) showed only slightly reduced neutralizing activity,^8–10^ while the other three VOCs (Beta-B.1.351, Gamma-P.1 and Delta-B.1.617.2) and four VOIs (Eta-B.1.525, Iota-B.1.526, Kappa-B.1.617.1 and Lambda-C.37) all displayed clear neutralization escape,^11–20^ of which the Beta variant was the most obvious, with approximately 3–10-fold reduction of neutralizing activity in convalescent sera or vaccine-immunized sera.^11–15^ Since April 2021, the Delta variant has caused a new wave of severe pandemic illness worldwide and has become the dominant SARS-CoV-2 strain. Subsequently, additional mutations occurred in the Delta variant (AY.1 and AY.2, named Delta plus variant), and this strain increased rapidly in the United States.^21,22^ The main difference between Delta plus and the original Delta variant was the addition of a K417N mutation in the receptor binding domain (RBD) of the S protein, which had also naturally occurred in other variants. Because the RBD is the key domain that determines binding affinity of virus to host cells, accumulation of mutations in this domain may cause changes in antigenicity. Currently, the top 10 current mutations in the RBD are N501Y, E484K, S477N, K417T, N439K, K417N, S494P, E484Q, F490S, and A520S (GISAID). The high mutation rate implies that these represent easily mutated sites that may be readily integrated into the current predominate variant, Delta strain, and thus further change the antigenicity of this VOC.

Altered antigenicity of SARS-CoV-2 variants implies changing effectiveness of vaccines. Many studies tried to develop vaccines by simply replacing original vaccine strains with new variants on their own platform.^23^ Whether vaccines should be updated following the emergence of variants is an important question that must be addressed. The WHO has established a very successful global surveillance and vaccine recommendation program for influenza (Global Influenza Surveillance and Response System, GISRS). Using this system, representative influenza strains from different countries are shared with the WHO collaborating Center for Influenza, and are then further analysed and reviewed. Antigenicity of the virus, especially the cross-neutralization between different strains, together with epidemiologic and disease information are used to form the evidence base for decisions on seasonal vaccine virus selection and candidate vaccine virus development, which are recommended by the WHO twice a year for the next winter in the southern and northern hemispheres, respectively. Similarly, the WHO Global Initiative on Sharing All Influenza Data (GISAID) also established the platform to monitor SARS-CoV-2 mutations worldwide; however, whether the same strategy for updating vaccine strains should be used for SARS-CoV-2 remains an issue to be urgently considered.

In the present study, we tested the neutralization activity of sera from animals immunized with the S protein from different VOCs against pseudotyped viruses containing naturally occurring VOCs and VOIs, as well as 20 possible variants based on the globally prevalent Delta variant. The antigenicity of the potential variants was analysed using convalescent- and vaccine-elicited sera. Meanwhile, we also tested sera from recombinant haemagglutinin (HA)-immunized animals for neutralization activity against H3N2 vaccine strains used from 2011–2019. The degree of cross-neutralizing activity and the structural difference of the antigenic variation between SARS-CoV-2 variants and H3N2 variants were also compared. This study provides important evidence and clues to predict whether the SARS-CoV-2 vaccine should be updated frequently like influenza.

## Results

### Construction of pseudotyped viruses for possible variants based on the Delta variant and the top 10 naturally occurring mutations in the RBD

**Fig. 1:**
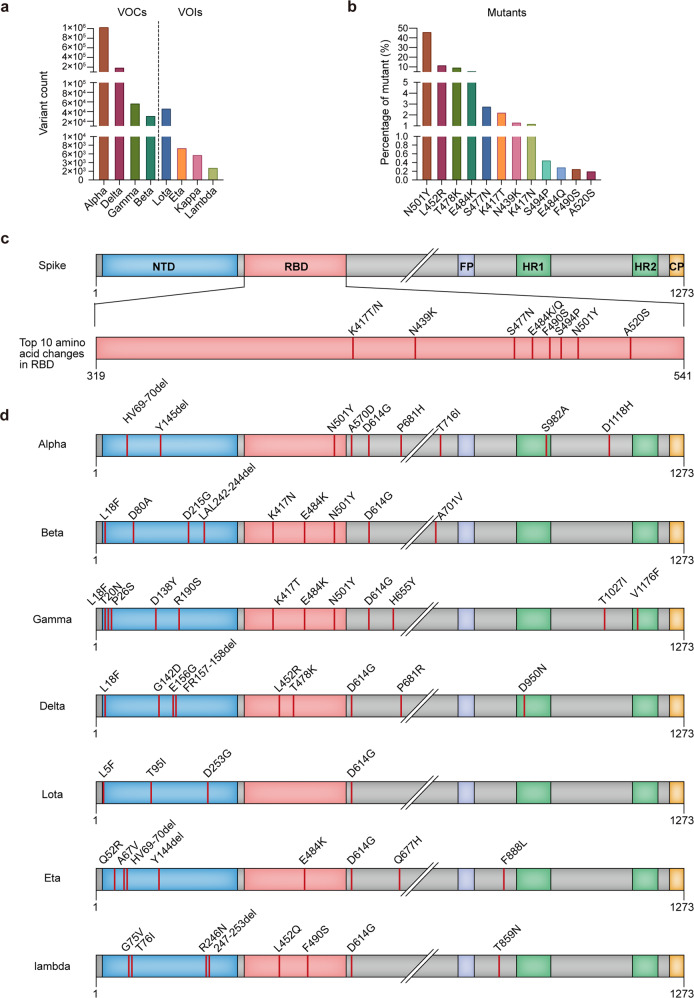
Circulating variants and most common amino acid changes in SARS-CoV-2 S protein. **a** VOC and VOI count. The VOC and VOI count data were obtained from outbreak.org (https://outbreak.info/situation-reports?name) up to July 19th, 2021. VOCs and VOIs were classified by the WHO. **b** The percentage of the top 12 amino acid changes in S protein. These data were sourced from COVIDCG.org (https://covidcg.org/?tab=home&undefined=All&selectedGene=S&residueCoordinates=319,541&startDate=2019-12-15&undefined=All) up to July 19th, 2021. **c** The amino acid changes introduced in this study. The most common amino acid changes except for L452R and T478K are indicated. (d) The illustration of the VOCs and VOIs. Delta was used as the template on which to add mutations. L452R and T478K located in the RBD of B.1.617.2 were not included in the additional mutagenesis. S spike, SARS-CoV-2 severe acute respiratory syndrome coronavirus 2, VOC variant of concern, VOI variant of interest, WHO World Health Organization, COVID coronavirus disease

The prevalence of VOCs and VOIs (Fig. 1a), and the top ten most common mutations located in the RBD region (Fig. 1b) were analysed. To predict antigenicity changes of potential future variants, we added the top 10 high-rate RBD amino acid changes individually and cumulatively (Fig. 1c) to the highly transmissible Delta variant. The amino acid changes of VOCs and VOIs were shown in Fig. 1d. Arranged in descending order, we firstly constructed 10 possible variants with single high-rate mutations based on the Delta strain, named D1 (N501Y), D2 (E484K), D3 (S477N), D4 (K417T), D5 (N439K), D6 (K417N), D7 (S494P), D8 (E484Q), D9 (F490S), and D10 (A520S). We then added a combination of high-rate mutations sequentially in the same order to generate another nine possible variants (Supplementary Fig. 1). It should be noted that D2 (E484K) and D8 (E484Q) located in the same position could not be introduced in the same variant, likewise for D4 (K417T) and D6(K417N), and thus we constructed four possible variants including all the mutation sites with different combinations of these mutations. In addition to the above variants, D614G, Delta, and two Delta plus variants were included in this study.

### Neutralization activity of Alpha, Beta, Gamma, and Delta S protein-elicited sera against VOCs and VOIs

**Fig. 2:**
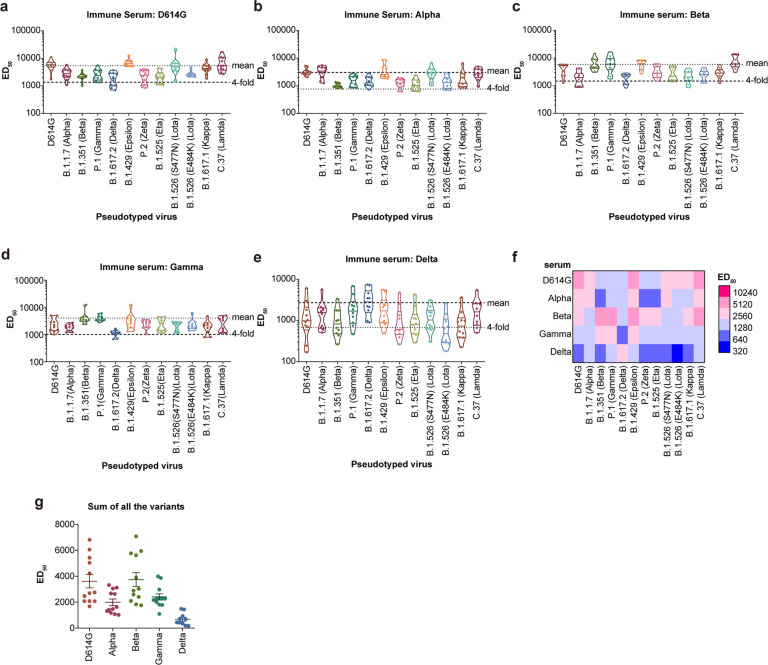
Neutralization activity of Alpha, Beta, and Gamma S protein-immunized sera against VOCs and VOIs. The SARS-CoV-2 S protein from D614G, Alpha, Beta, or Gamma variants were used to immunize mice. The sera were collected for neutralization activity testing against the indicated VOCs and VOIs. The mean results of neutralization ED_50_ from three repeated experiments are shown as a dot plot. **a**–**d** The red arrow indicates neutralization against homologous pseudotyped virus. The dashed lines represent the geometric mean and fourfold reduction for serum response of the homologous virus, respectively. **e** ED_50_ heat map of homologous and heterologous sera and virus. **f** ED_50_ of one serum against homologous and heterologous variants are summarized into one column. S spike, VOC variant of concern, VOI variant of interest, ED_50_, 50% effective dilution

To study the immunogenicity of the S protein of different variants, we immunized mice with the S protein trimer from Alpha, Beta, Gamma, Delta, or D614G. The neutralization activity of the immunized sera was tested against pseudotyped VOCs and VOIs. We found that most sera showed highest inhibition against the homologous pseudotyped viruses (Fig. 2a–d). However, differences in neutralization sensitivity of a particular serum against heterologous variants were relatively small, all less than fourfold compared with that of homologous virus (Fig. 2a–e). When we compared the neutralization ability of heterologous immune sera to particular pseudotyped viruses, changes in neutralization ability were also within fourfold (Fig. 2e). Furthermore, although the titer of Beta or Gamma S protein-elicited sera against the Beta and Gamma variants was much higher than that of D614G or Alpha S protein-elicited sera, the titres against other variants (e.g., Alpha and D614G variants) were lower (Fig. 2f). When we summarized the neutralization titres from all the variants, titres of the sera from D614G and Beta S protein-immunization were higher than those of Alpha and Gamma variants (Fig. 2g). Therefore, when we compared Alpha, Beta or Gamma, we could not determine which variant was more suitable than another to become a candidate new vaccine to replace the currently available original vaccine strain.

### Neutralization activity of Alpha, Beta, Gamma, and Delta S protein-elicited sera against possible Delta variants

**Fig. 3:**
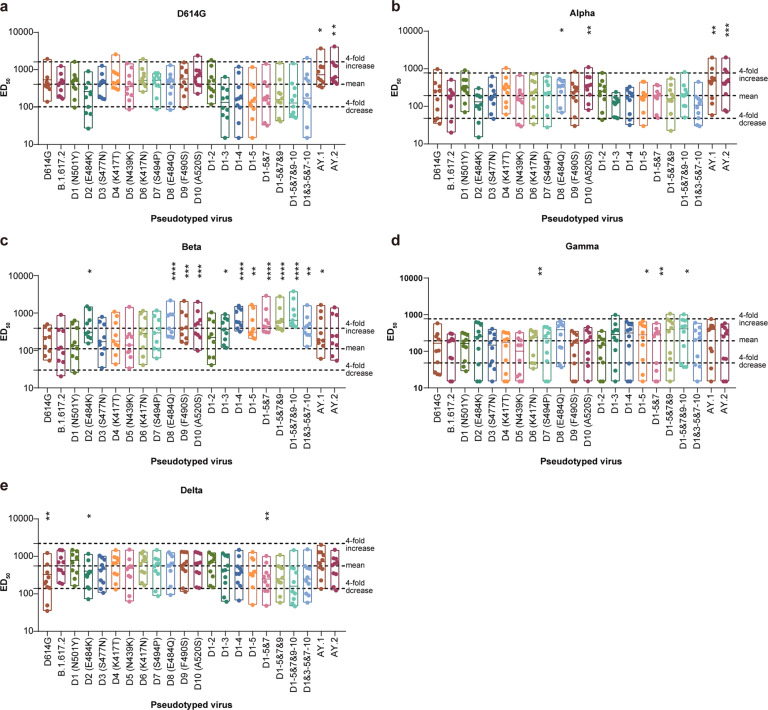
Neutralization activity of Alpha, Beta, and Gamma S protein- immunized sera against possible Delta variants with multiple amino acid changes in RBD. The S protein trimer from D614G and three VOCs (Alpha, Beta, Gamma) were used individually to immunize mice. Sera were collected and tested for neutralization activity against B.1.617.2 (Delta) variant-based possible variants with multiple amino acid changes in RBD. Mean ED_50_ from three repeated experiments are shown as dot plots. The dashed lines represent the geometric mean and fourfold reduction for serum response of the reference Delta variant, respectively. Dashed lines indicate a fourfold difference of ED_50_ compared with the Delta reference strain. S spike, RBD receptor binding domain, SARS-CoV-2 severe acute respiratory syndrome coronavirus 2, VOC variant of concern, ED_50_ 50% effective dilution

The top 10 mutations in the RBD represent a natural hot-spot of amino acid changes and might combine with currently circulating variants. To investigate whether accumulating amino acid changes in Delta would further increase the possibility of immune escape, we used immunized sera from Alpha, Beta, Gamma, Delta, and D614G S protein-immunized animals to test the antigenicity of possible variants with RBD hot-spot amino acid changes on the Delta variant background. When a single mutation was added, no obvious neutralization decrease was observed in any of the four sera. With accumulation of mutations, the sensitivity of the pseudotyped potential variants to neutralization by D614G and Alpha sera decreased gradually. However, these decreases in sensitivity remained less than fourfold (Fig. 3a and b). Conversely, when mutations were accumulated, the sensitivity of the pseudotyped possible variants to neutralization by Beta, Gamma, and Delta sera increased gradually (Fig. 3c–e). This trend was particularly obvious in the Beta sera, where more than fourfold increases were observed in some variants (Fig. 3c). This may be explained by the fact that many of the added mutations, such as N501Y, K417T/N, and E484K/Q, are present in the Beta variant. These results also show that the antigenicities of D614G, Alpha and Delta are similar, while those of Beta and Gamma are different.

### Neutralization activity of convalescent- and vaccine-elicited sera against possible Delta variants

**Fig. 4:**
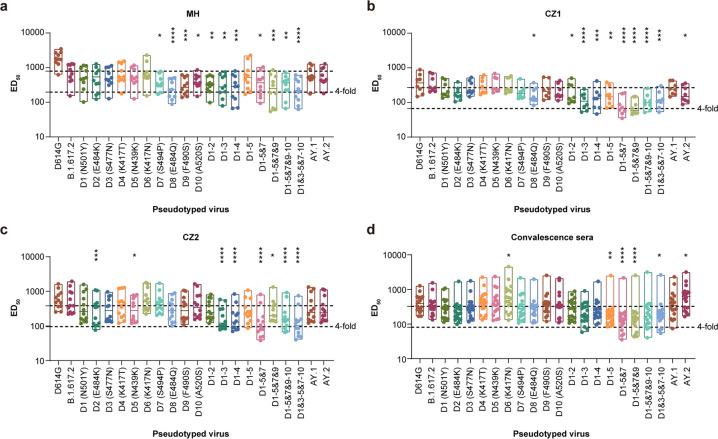
Neutralization activity of convalescent and vaccine-elicited sera against possible Delta variants with multiple amino acid changes in RBD. The neutralization activity of convalescent serum (**a**), recombinant S protein vaccine-immunized sera (**b** and **c**), and inactive vaccine-immunized sera (**d**) against different potential SARS-CoV-2 variants were tested. Mean ED_50_ from three repeated experiments are shown as dot plots. The dashed lines represent the geometric mean and fourfold reduction for serum response of the reference Delta variant, respectively. RBD receptor binding domain, SARS-CoV-2 severe acute respiratory syndrome coronavirus 2, ED_50_ 50% effective dilution

To further illustrate the neutralization effect of SARS-CoV-2 vaccines on possible Delta variants with cumulative amino acid changes, we examined clinical immune sera following vaccination with either inactivated vaccine or one of two recombinant protein vaccines (Fig. 4). We found that with the accumulation of amino acid changes in the RBD, neutralization activity induced by the inactivated vaccine was somewhat reduced (less than fourfold, Fig. 4a). Immune sera following recombinant vaccine immunization showed a similar trend but was more obviously decreased with pseudotyped viruses, D1-5&7&9 and D1-5&7, to slightly more than a fourfold reduction (Fig. 4b and c). Compared with the inactivated vaccine, which uses the whole SARS-CoV-2 particle as immunogen, these recombinant vaccines used the dimeric RBD as the immunogen, thus accumulated mutations in this region may cause more obvious changes in sensitivity to the recombinant vaccine-induced neutralization. Similar to the immune serum induced by the inactivated vaccine, the neutralization activity of convalescent sera against possible Delta variants with multi-site mutations also decreased, although to a slightly lesser extent than that of inactivated or recombinant vaccine immune sera (Fig. 4d).

### Neutralization activity of influenza HA plasmid-immunized serum against H3N2 vaccine strains

**Fig. 5:**
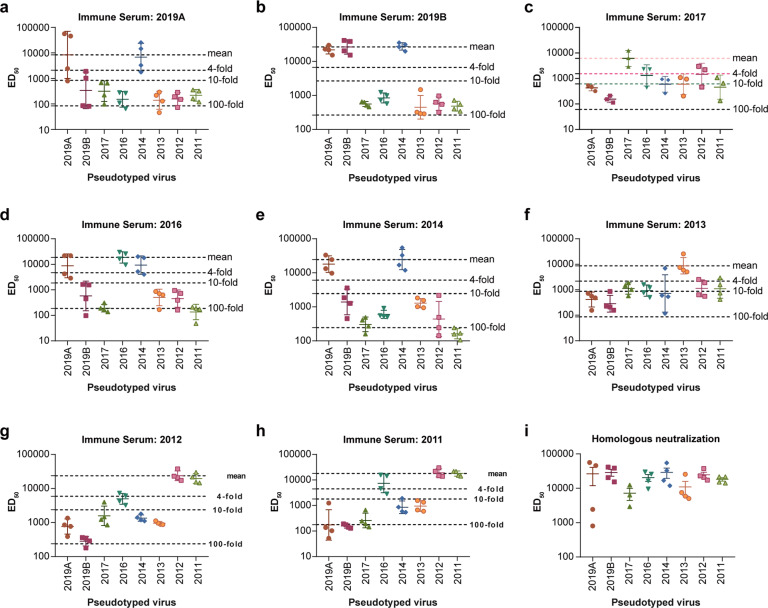
Neutralization activity of influenza HA plasmid-immunized sera against H3N2 vaccine strains. Guinea pigs were immunized with influenza HA plasmids of the vaccine strains of 2011–2019. Sera were collected for neutralization activity testing against pseudotyped viruses of the H3N2 vaccine strains from 2011–2019. Mean ED_50_ from three repeated experiments are shown as dot plots. Dashed lines indicate 4-, 10-, and 100-fold differences compared with the corresponding vaccine strain. The homologous neutralization activity is compared in the last graph. HA haemagglutinin, H3N2 haemagglutinin 3 neuraminidase 2, ED_50_ 50% effective dilution

To compare the impact antigenicity changes of SARS-CoV-2 variants and H3N2 seasonal influenza variants, we analysed the antigenicity differences of H3N2 seasonal influenza vaccine strains in different years (2012–2019) using the same pseudotyped virus technique. The results showed that immune sera of most vaccine strains could not neutralize pseudotyped viruses containing the HA of the following year. The reductions in neutralization sensitivity were always more than tenfold, in some cases even more than 100-fold (e.g., 2011 serum against 2016 virus), which was dramatically greater than the effects of antigenicity changes observed in our SARS-CoV-2 pseudotyped virus system (Fig. 5).

### Antigenic variation analyses of H3N2 HA and SARS-CoV-2 S proteins

**Fig. 6:**
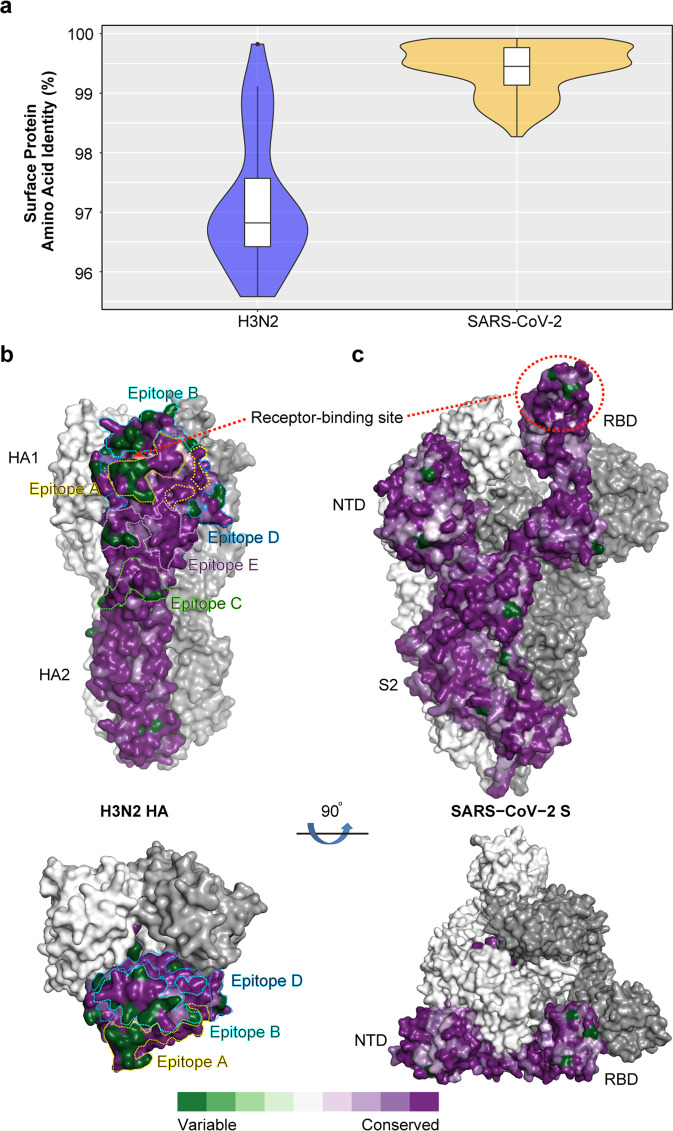
Antigenic variation analyses of H3N2 HA and SARS-CoV-2 S proteins. **a** The percent identities of amino acid sequences of representative H3N2 HA and SARS-CoV-2 S protein are shown respectively. **b** The surface view of an H3N2 HA trimer (A/Victoria/361/2011; PDB code: 4O5N) is shown. One monomer of the HA surface is colored using the ConSurf server^34^ according to sequence conservation from the most conserved (dark purple) to the most divergent (dark green) based on the alignment of H3N2 sequences from different vaccine strains. The five antigenic sites A (yellow), B (cyan), C (green), D (blue) and E (pink) are highlighted in dotted circles. **c** The surface view of the SARS-CoV-2 D614G S protein trimer (PDB code: 7KE9) is shown. One monomer of the S surface is colored using the ConSurf server^34^ according to sequence conservation from the most conserved (dark purple) to the most divergent (dark green) based on the alignment of S sequences from different VOC strains. The receptor-binding sites are highlighted in dotted circles. HA haemagglutinin, H3N2 haemagglutinin 3 neuraminidase 2, SARS-CoV-2 severe acute respiratory syndrome coronavirus 2, S spike, PDB protein data bank, VOC variant of concern

Consistent with the experiment above, the antigenic variation analysis also showed that H3N2 viruses are more genetically divergent than SARS-CoV-2, with lower sequence identities in the major antigenic protein, HA (Fig. 6a). Structural analysis showed that the most variable region of H3N2 HA (A/Victoria/361/2011; PDB code: 4O5N) was located in the globular region, consisting of five classic antigenic sites (Fig. 6b), especially sites A and B near the receptor binding region, while the most variable regions of SARS-CoV-2 S were located in the RBD and NTD regions (PDB code: 7KE9, Fig. 6c). Regions around the receptor binding sites of both HA and S surface proteins showed diverse features indicating that these regions are hot-spots for antibody escape. More amino acid substitutions were observed in HA at these antigenic sites compared with the number of substitutions in the SARS-CoV-2 S protein, which may be the reason for the observed increase in antigenicity differences in H3N2 viruses.

## Discussion

The COVID-19 pandemic has been spreading globally for almost two years. Based on prior academic and industry research on SARS, MERS, and other related coronaviruses, several SARS-CoV-2 vaccines have been successfully developed within a very short time. To date, there are 24 vaccines within the WHO Emergency Use Listing/Pre-qualification (EUL/PQ) evaluation phase, and 299 vaccines are in clinical or preclinical development, including mRNA-, adenovirus-, recombinant S/RBD protein-, and inactivated whole virus vaccines 24. Over 5 billion vaccinations have been administered worldwide by 14th September (https://www.who.int/). With the ongoing increase in vaccine administration, the infection rate of SARS-CoV-2 in many countries has decreased significantly.^24^ However, the emergence of SARS-CoV-2 variants has led to the emergence of breakthrough infections, especially the recent emergence of Delta variants that have been detected in 170 countries. A recent report showed that even in Israel, the country with the highest levels of vaccination, the Delta variant caused a sharp increase in infections.^25^ Therefore, whether the original strain used in vaccines should be changed has become an important question.

Although both cellular and humoral immune responses play important roles in preventing COVID-19 infection, it is generally believed that the higher the titer of neutralizing antibodies, the better the protective effect against COVID-19.^26,27^ In this study, using Alpha-, Beta-, Gamma- and Delta-derived S protein-immunized animal sera, the neutralization activities were mostly reduced within fourfold against the current VOCs and VOIs. Even if the top 10 RBD mutations were added independently or cumulatively to the Delta variant, the changes in antigenicity of the possible variants remained within four folds. This study indicated that different VOCs, VOIs, and possible variants of SARS-CoV-2 showed high degrees of cross-neutralization. If a candidate vaccine induces high levels of neutralizing antibody, it should protect against infection with all variants. Conversely, the antigenicity among influenza virus H3N2 strains was significantly different, up to 10–1000-fold. In terms of antigenicity, the differences caused by SARS-CoV-2 mutations were much lower than those of seasonal influenza viruses between different epidemic seasons. However, with the increasing number of circulating variants, the immune response induced by vaccines based on the original strain of SARS-CoV-2 were decreased to a certain extent, although most of the vaccines remain protective. It should be noted that the protective immune response of effective SARS-CoV-2 vaccine includes not only neutralizing antibodies but also long-lived memory B cells and memory T cells. Memory B cells and memory T cells are essential for evaluating the persistence of vaccine-induced immune responses. It is reported that memory B cells and memory T cells induced by mRNA vaccine have a good correlation with humoral immunity.^28^ The magnitude of the neutralizing antibody level could partially predict the durability of the protective immune responses induced by the SARS-CoV-2 vaccines. Despite the threat of current and future variants,^29^ replacement of the vaccine strain for COVID-19 vaccines may not be as urgent as we suspected but should remain under surveillance, with how to upgrade the vaccine requiring careful consideration.

Three strategies should be considered for the update of vaccines. First, the structure of the immunogen should be optimized to improve its ability to induce higher neutralizing antibodies, cellular immune responses, and immune memory no matter which variants are used. Importantly, there are conserved epitopes in the SARS-CoV-2 S protein, and epitope changes to these sites will significantly affect the fitness of the virus,^30^ making immune escape difficult. Vaccines can be designed to target these conserved regions to ensure that immune protective effects are broad-spectrum or potentially even pan-coronavirus. Second, the immune doses of existing vaccines need further optimization to balance safety and effectiveness, to avoid the decline in protective effect caused by insufficient dosage while also minimizing safety risks caused by high doses. Thirdly, the effectiveness and persistence of immune protection will be further improved by optimizing the immune strategy. Through booster immunizations, the immune response induced by the vaccine could be further enhanced. However, the timing and dose of booster need further investigation. Besides, through heterologous immunization with different types of vaccines, the range of immune responses induced by various vaccines may be diversified, thus enriching the protective immune response.

In conclusion, our findings suggest that using a specific current variant to replace the original vaccine strain may not be necessary at this time. The optimal design of SARS-CoV-2 vaccines should be comprehensively investigated through the optimization of immunogen, dose, and immunization strategy, while monitoring changes in antigenicity. With continuing emerging of new variant, close monitoring and functional analysis of them could be informative for guiding prevention and control measures for the SARS-CoV-2 pandemic.

## Materials and methods

### Generation and production of pseudotyped SARS-CoV-2 variants

The SARS-CoV-2 spike (GenBank: MN908947) protein expression gene was optimized using a mammalian codon and cloned into the pcDNA3.1 vector. Site-directed mutagenesis was performed as described previously.^31^ Specific mutation sites and corresponding primers are presented in Supplementary Table 1. 293 T cells were transfected with S protein expression plasmids and simultaneously infected with G*ΔG-VSV (Kerafast, Boston, MA). The supernatant containing the pseudotyped virus was harvested 24 h and 48 h later.

### Generation and production of pseudotyped H3N2 influenza viruses

The H3N2 influenza virus HA protein expression plasmids were constructed by inserting HA genes into the pcDNA3.1. The HA genes uses in this study were sourced from GISAID, including A/Hong Kong/45/2019 (EPI1691930), A/Hong Kong/2671/2019 (EPI1884455), A/Kansas/14/2017 (EPI1868579), A/Singapore/INFIMH-16-0019/2016 (EPI1858150), A/Hong Kong/4801/2014 (EPI1868553), A /Switzerland/9715293/2013 (EPI1868576), A/Texas/50/2012 (EPI1868581), and A/Victoria/361/2011 (EPI1868575). 293 T cells were transfected with HA protein expression plasmids and simultaneously infected with G*ΔG-VSV. After 4 h of transfection, neuraminidase from *Clostridium* (Aladdin, Shanghai, China) was added to release the newly-packaged pseudotyped viruses. The supernatant containing the pseudotyped viruses was harvested 24 h later.

### Titration of SARS-CoV-2 and H3N2 influenza pseudotyped viruses

The titer of pseudotyped viruses was evaluated by infecting Huh 7 cells with threefold serial dilutions of pseudotyped virus. H3N2 influenza pseudotyped viruses were activated by addition of 1.0 mg/mL (TPCK)-trypsin (Sigma-Aldrich, St Louis, MO) to each threefold diluted well, to a final concentration of 40 µg/mL. The plate was incubated at 37 °C with 5% CO_2_, and after 30 s, Huh7 cells (Japanese Collection of Research Bioresources [JCRB]0403; 2 × 10^4^ cells/well) were added to each well of the 96-well titration plate. After 24 h incubation at 37 °C with 5% CO_2_, chemiluminescence signals were detected using the Britelite plus reporter gene assay system (PerkinElmer, Waltham, MA).

### Sera from SARS-CoV-2 convalescent patients

Serum samples from SARS-CoV-2 convalescent patients were provided by Dr. Xiaowang Qu from the University of South China. Eighteen samples were collected from patients in Hunan who had been infected with SARS-CoV-2 (WH-1 reference strain). The sera were collected 14–28 days after discharge. Written informed consent was obtained from all patients prior to blood collection. The study protocol involving convalescent serum samples was approved by the Ethics Committee of the Chinese Center for Disease Control and Prevention.

### Sera from vaccinated individuals

For the group of patients receiving inactivated virus vaccine (MH), serum samples were provided by Dr Jiankai Liu, of the Shenzhen Kangtai Biological Products Co., China. Samples were collected from ten individuals who had been immunized with inactivated virus vaccine (KCONVAC, Shenzhen Kangtai Biological Products Co.; Chinese Clinical Trial Registry: ChiCTR2000038804) at 14 days after the completion of a standard immunization procedure (at 0, 28, and 58 days; 5 µg/dose). Written informed consent was obtained from all volunteers prior to blood collection. The study protocol involving the inactivated virus vaccine was approved by the Ethics Committee of Jiangsu Provincial Center for Disease Control and Prevention.

Sera from individuals vaccinated with recombinant vaccine were provided by Anhui Zhifei Longcom Biopharmaceutical Co., Ltd, and Liaoning Mao Kang Yuan Biotech Co. Ltd., China. For the first group receiving the recombinant vaccine (CZ-1), serum samples were collected from eight individuals who had been immunized with three doses (at 0, 1, and 2 months) of vaccine at 25 µg/dose (Recombinant Novel Coronavirus Vaccine [CHO cell], Anhui Zhifei Longcom Biopharmaceutical Co., Ltd, ClinicalTrials.gov Identifier: NCT04466085). Samples were collected at 30 days after the third immunization. Written informed consent was obtained from all volunteers prior to blood collection. The study protocol was approved by the Ethics Committee of Hunan Provincial Center for Disease Control and Prevention.

For the second group receiving recombinant vaccine (CZ-2), serum samples were collected from twelve individuals who had been immunized with 3 doses (at 0, 1, and 2 months) of vaccine at 20 µg/dose or 40 µg/dose (Recombinant SARS-CoV-2 Vaccine [CHO cell], Liaoning Mao Kang Yuan Biotech Co. Ltd., ClinicalTrials.gov Identifier: NCT04813562). Samples were collected at 30 days after the third immunization. Written informed consent was obtained from all volunteers prior to blood collection. The study protocol was approved by the Ethics Committee of Jiangsu Provincial Center for Disease Control and Prevention.

### Sera from mice immunized with purified S protein of SARS-CoV-2 variants

The SARS-CoV-2 S protein expressed from human HEK293 cells in trimeric prefusion state were obtained from Acrobiosystems Inc, Beijing, China. The S protein used in this study included D614G (cat# SPN-C52H3), Alpha (cat# SPN-C52H6), Beta (cat# SPN-C52Hk), Gamma (cat# SPN-C52Hg), and Delta (cat# SPN-C52He). Female BALB/c mice aged 6–8 weeks were immunized subcutaneously with 20 µg purified S protein with 100 µg Aluminum Phosphate Adjuvant (once every 7 days for three inoculations). Blood samples were collected 7 days after the third immunization. Mice were handled under institutional (NIFDC, Beijing, China) guidelines for laboratory animal care and use, and the Animal Care and Use Committee at the NIFDC approved the animal study protocol.

### Sera from guinea pigs immunized with H3N2 vaccine strain HA plasmid

Female guinea pigs (four per group, body weight 200–220 g) were inoculated with 200 µg of one of the following eight plasmids: pcDNA3.1-A/Hong Kong/2671/2019 HA, pcDNA3.1-A/Hong Kong/45/2019 HA, pcDNA3.1-A/Kansas/14/2017 HA, pcDNA3.1-A/Singapore/INFIMH-16-0019/2016, pcDNA3.1-A /Switzerland/9715293/2013(2013), pcDNA3.1-A/Hong Kong/4801/2014 HA, pcDNA3.1-A/Switzerland/9715293/2013 HA, pcDNA3.1-A/Texas/50/2012 HA, and pcDNA3.1-A/Victoria/361/2011 HA. Immunization was performed three times at fortnightly intervals, and sera were collected 2 weeks after the third immunization. Guinea pigs were handled under institutional (NIFDC, Beijing, China) guidelines for laboratory animal care and use, and the Animal Care and Use Committee at the NIFDC approved the animal study protocol.

### In vitro neutralization assay with pseudotyped viruses

For the neutralization assays, serial dilutions (starting at 1:30) of serum samples were mixed with 1.3 × 10^4^ TCID_50_ of pseudotyped SARS-CoV-2 variants or H3N2 influenza pseudotyped viruses in 96-well plates at 37 °C for 1 h, then mixed with Huh 7 cells and subsequently incubated for 24 h. The infectivity was determined by measuring the bioluminescence as described previously.^32^ The 50% effective dilution (ED_50_) was calculated using the Reed–Muench method. The results were recorded as the mean of three replicates.

### Antigenic variation analyses of H3N2 HA and SARS-CoV-2 S proteins

The sequences of antigens from representative H3N2 vaccine strains and SARS-COV-2 VOC strains were chosen for further analysis. To describe the genetic diversity between sequences, we calculated the p-distance between each pair of amino acid sequences in H3N2 HA and SARS-COV-2 S proteins (additionally including the Wuhan-Hu-1 isolate that was used to design the current COVID-19 vaccines) in MEGA X.^33^ We then converted the p-distance into the percent identity using the formula, $(1 - {{{\mathrm{p - distance}}}}) \times 100$. Structure figures were prepared using PyMOL (http://pymol.org/).

### Statistical analysis

GraphPad Prism 8 (GraphPad, San Diego, CA) was used for statistical analysis. One-way ANOVA and Holm–Sidak multiple comparisons tests were used for comparisons. Values are shown as means ± standard error of the mean (SEM). Significance thresholds were as follows: *p < 0.05, **p < 0.01, ***p < 0.001, and ****p < 0.0001.

## Supplementary information

Supplementary material

## Data Availability

Original data for Figs. 2, 3, 4, and 5 have been deposited to figshare: https://figshare.com/articles/dataset/The_antigenicity_of_SARS-CoV-2_Delta_variants_aggregated_10_high-frequency_mutations_in_RBD_has_not_changed_sufficiently_to_replace_the_current_vaccine_strain/17134154

## References

[1] Lu R (2020). Genomic characterisation and epidemiology of 2019 novel coronavirus: implications for virus origins and receptor binding. Lancet.

[2] Kim D (2020). The architecture of SARS-CoV-2 transcriptome. Cell.

[3] Yao H (2020). Molecular architecture of the SARS-CoV-2 virus. Cell.

[4] Forni G, Mantovani A, Covid-19 Commission of Accademia Nazionale dei Lincei, R. (2021). COVID-19 vaccines: where we stand and challenges ahead. Cell Death Differ..

[5] Snijder EJ (2020). A unifying structural and functional model of the coronavirus replication organelle: Tracking down RNA synthesis. PLoS Biol..

[6] Smith EC, Denison MR (2013). Coronaviruses as DNA wannabes: a new model for the regulation of RNA virus replication fidelity. PLoS Pathog..

[7] Denison MR (2011). Coronaviruses: an RNA proofreading machine regulates replication fidelity and diversity. RNA Biol..

[8] Wu J (2021). The Antigenicity of Epidemic SARS-CoV-2 Variants in the United Kingdom. Front Immunol..

[9] Muik, A. et al. Neutralization of SARS-CoV-2 lineage B.1.1.7 pseudovirus by BNT162b2 vaccine–elicited human sera. *Science* **371**, 1152–1153 (2021). 10.1126/science.abg6105 PMC7971771 33514629

[10] Garcia-Beltran WF (2021). Multiple SARS-CoV-2 variants escape neutralization by vaccine-induced humoral immunity. Cell.

[11] Zhang L (2021). Ten emerging SARS-CoV-2 spike variants exhibit variable infectivity, animal tropism, and antibody neutralization. Commun. Biol..

[12] Edara VV (2021). Infection- and vaccine-induced antibody binding and neutralization of the B.1.351 SARS-CoV-2 variant. Cell Host Microbe.

[13] Li Q (2021). SARS-CoV-2 501Y.V2 variants lack higher infectivity but do have immune escape. Cell.

[14] Wang P (2021). Antibody resistance of SARS-CoV-2 variants B.1.351 and B.1.1.7. Nature.

[15] Planas D (2021). Sensitivity of infectious SARS-CoV-2 B.1.1.7 and B.1.351 variants to neutralizing antibodies. Nat. Med.

[16] Zhang, L. et al. Infectivity and antigenicity of SARS-CoV-2 B.1.617 variants. *Res. Square*, 10.21203/rs.3.rs-596463/v1 (2021).

[17] Liu C (2021). Reduced neutralization of SARS-CoV-2 B.1.617 by vaccine and convalescent serum. Cell.

[18] West AP (2021). Detection and characterization of the SARS-CoV-2 lineage B.1.526 in New York. Nat. Commun..

[19] Tada, T. et al. SARS-CoV-2 Lambda variant remains susceptible to neutralization by mRNA vaccine-elicited antibodies and convalescent serum. 2021.2007.2002.450959 (2021).

[20] Kimura, I. et al. SARS-CoV-2 Lambda variant exhibits higher infectivity and immune resistance. 2021.2007.2028.454085 (2021). 10.1016/j.celrep.2021.110218 PMC8683271 34968415

[21] Bolze, A. et al. SARS-CoV-2 variant Delta rapidly displaced variant Alpha in the United States and led to higher viral loads. 2021.2006.2020.21259195 (2021). 10.1016/j.xcrm.2022.100564 PMC8922438 35474739

[22] Kannan SR (2021). Evolutionary analysis of the Delta and Delta Plus variants of the SARS-CoV-2 viruses. J. Autoimmun..

[23] Choi, A. et al. Safety and immunogenicity of SARS-CoV-2 variant mRNA vaccine boosters in healthy adults: an interim analysis. *Nat. Med.* **27**, 2025–2031 (2021). 10.1038/s41591-021-01527-y PMC8604720 34526698

[24] Subbarao K (2021). The success of SARS-CoV-2 vaccines and challenges ahead. Cell Host Microbe.

[25] Wadman M (2021). Israel’s grim warning: delta can overwhelm shots. Science.

[26] Gilbert, P. B. et al. Immune correlates analysis of the mRNA-1273 COVID-19 vaccine efficacy clinical trial. *Science*, **375**, 43–50 (2022). 10.1126/science.abm3425 PMC9017870 34812653

[27] Corbett KS (2021). Immune correlates of protection by mRNA-1273 vaccine against SARS-CoV-2 in nonhuman primates. Science.

[28] Goel RR (2021). mRNA vaccines induce durable immune memory to SARS-CoV-2 and variants of concern. Science.

[29] He, X. et al. SARS-CoV-2 Omicron variant: characteristics and prevention. *MedComm* **16**, 838–845 (2021). 10.1002/mco2.110 PMC8693031 34957469

[30] Schmidt F (2021). High genetic barrier to SARS-CoV-2 polyclonal neutralizing antibody escape. Nature.

[31] Nie J (2020). Establishment and validation of a pseudovirus neutralization assay for SARS-CoV-2. Emerg. Microbes Infect..

[32] Li Q (2020). The impact of mutations in SARS-CoV-2 spike on viral infectivity and antigenicity. Cell.

[33] Kumar S (2018). MEGA X: molecular evolutionary genetics analysis across computing platforms. Mol. Biol. Evol..

[34] Ashkenazy H (2016). ConSurf 2016: an improved methodology to estimate and visualize evolutionary conservation in macromolecules. Nucleic Acids Res..

