# Supplementary material for: The antigenicity of SARS-CoV-2 Delta variants aggregated 10 high-frequency mutations in RBD has not changed sufficiently to replace the current vaccine strain
Source: Signal Transduct Target Ther. 2022 Jan 19;7:18. doi: 10.1038/s41392-022-00874-7 (PMC8767530; doi:10.1038/s41392-022-00874-7)
Supplement: Supplementary file 1 — Supplementary material [file 41392_2022_874_MOESM1_ESM.docx]

Supplementary Materials for

**The antigenicity of SARS-CoV-2 Delta variants aggregated 10 high-frequency mutations in RBD has not changed sufficiently to replace the current vaccine strain**

Jiajing Wu, Jianhui Nie, Li Zhang, Hao Song, Yimeng An, Ziteng Liang, Jing Yang, Ruxia Ding, Shuo Liu, Qianqian Li, Tao Li, Zhimin Cui, Mengyi Zhang, Peng He, Youchun Wang, Xiaowang Qu, Zhongyu Hu, Qihui Wang, and Weijin Huang

Correspondence to: huzhy@nifdc.org.cn, wangqihui@im.ac.cn, [huangweijin@nifdc.org.cn](mailto:huangweijin@nifdc.org.cn)

**This file includes:**

**Figure S1. SARS-CoV-2 variants pseudotyped viruses constructed in this communication.**

**Table S1. Primes used to do the site-directed mutagenesis**

| **Oligonucleotides** | SOURCE |
| --- | --- |
| VSV (P protein)-F: TCTCGTCTGGATCAGGCGG | SinoGenoMax Company Limited |
| VSV (P protein)-R: TGCTCTTCCACTCCA GENEWIZ TCCTCTTGG | SinoGenoMax Company Limited |
| T19R-F:  CAGCCAGTGCGTGAATCTGAGAACCAGAACCCAGCTG | Sangon Biotech |
| V70F-F: CCTGGTTCCACGCCATCCACTTCAGCGGCACCAATGGCAC | SinoGenoMax Company Limited |
| T95I-F: GACGGCGTGTACTTCGCCAGCATCGAGAAGAGCAATATCATCAG | Sangon Biotech |
| G142D-F: CAATGACCCTTTCCTGGACGTTTATTATCATAAGAACAA | Sangon Biotech |
| EFR156-158G-F: CAAGAGCTGGATGGAGAGCGGGGTATATTCGTCGGCTAATAATTGCACC | SinoGenoMax Company Limited |
| A222V-F: CTGCCTCAGGGCTTCAGCGTGCTGGAGCCTCTGGTGGACC | Sangon Biotech |
| W258L-F: CAGGCGATTCGTCAAGCGGTCTGACCGCTGGAGCTGCGGCA | SinoGenoMax Company Limited |
| K417N-F: CGCCAGGGCAGACCGGCAATATCGCCGACTACAATTAC | Sangon Biotech |
| K417T-F:  CGCCAGGGCAGACCGGCACCATCGCCGACTACAATTAC | Sangon Biotech |
| N439K-F: CTGCGTGATCGCGTGGAACTCTAAGAACCTGGACTCGAAAGTTGGAGGC | Sangon Biotech |
| L452R-F: TTGGAGGCAATTACAATTACCGGTACAGACTGTTCAGAAAGAG | Sangon Biotech |
| S477N-F: GCACCGAGATCTACCAGGCCGGCAACACACCGTGTAATGGCGTGGAGGGC | Sangon Biotech |
| ST477-478NK-F: GCACCGAGATCTACCAGGCCGGCAACAAACCGTGTAATGGCGTGGAGGGC | Sangon Biotech |
| T478K-F: ATCTACCAGGCCGGCAGCAAACCGTGTAATGGCGTG | Sangon Biotech |
| E484Q-F: CACCGTGTAATGGCGTGCAGGGCTTCAATTGCTACTTC | Sangon Biotech |
| E484K-F: CGTGTAATGGCGTGAAGGGCTTCAATTGCTACTTCCCT | Sangon Biotech |
| F490S-F: GTGGAGGGCTTCAATTGCTACAGC CCTCTGCAGAGCTACGGC | Sangon Biotech |
| S494P-F: CAATTGCTACTTCCCTCTGCAGCCT TACGGCTTCCAGCCTACCA | Sangon Biotech |
| FPLQS490-494SPLQF-F: GTGGAGGGCTTCAATTGCTACAGC CCTCTGCAGCCTTACGGCTTCC | SinoGenoMax Company Limited |
| N501Y-F: AGAGCTACGGCTTCCAGCCTACCTACGGCGTGGGCTACCAGCCTTACAG | Sangon Biotech |
| A520S-F: TGCTGAGCTTCGAGCTGCTGCACAGCCCCGCTACCGTGTGCGGCCCTA | Sangon Biotech |
| K558N-F: CTGACCGAGAGCAATAAGAATTTTCTTCCCTTTCAACAAT | SinoGenoMax Company Limited |
| D614G-F: GTGGCCGTGCTGTACCAGGGCGTGAATTGCACCGAGGT | Sangon Biotech |
| P681R-F: CAGACCCAGACCAATAGCAGAAGAAGAGCCAGAAGC | Sangon Biotech |
| D950N-F: CTGGGCAAGCTGCAGAACGTGGTGAATCAGAATG | Sangon Biotech |
| V1228L-F: TCGCCGGCCTGATCGCCATCCTGATGGTGACCATCATGCTGTGCTG | SinoGenoMax Company Limited |
